# A rare case of symptomatic hydrometrocolpos in a 5y old female

**DOI:** 10.1016/j.eucr.2021.101789

**Published:** 2021-07-27

**Authors:** Hussam Nassar, Maya Horst, Rita Gobet

**Affiliations:** Division of Pediatric Urology, University Children's Hospital Zurich, Steinwiesstr. 75, 8032, Zurich, Switzerland

**Keywords:** Ectopic ureter, Dysplastic kidney, OHVIRA, Hydrometrocolpos

## Abstract

Late presentation of symptomatic hydrometrocolpos is uncommon. We present a 5 years old continent girl with prenatally diagnosed multicystic dysplastic left kidney and late-onset of lower abdominal pain. Investigations revealed a nonfunctioning left kidney with an ectopic ureter draining into the left hemivagina, and a vaginal duplication with an obstructed and urine-filled left hemivagina. Surgical therapy included resection of the vaginal septum and laparoscopic nephroureterectomy. Not only renal agenesis but also dysplasia or multicystic kidney may part of an OHVIRA syndrome. In girls with unilateral renal dysplasia, a duplication of the internal genitalia must always be considered.

## 1 Introduction

Ectopic ureters are considered as a rare entity. The occurrence of ectopic ureters is 1/2000 in newborns with a gender-ratio of 6:1 in favor of females. The majority (80%) of ectopic ureters are associated with a duplex renal system, whereas only 20% are seen in a single system.^1^ They are usually diagnosed prenatally or in the early postnatal period due to recurrent urinary tract infections in males or continuous incontinence in females.^1^ Unlike the duplex system, diagnosis of an ectopic ureter in a single system can be challenging and delayed because it mostly drains dysplastic and hypo/nonfunctional kidneys.^2^ Ectopic ureters drain frequently in the Müllerian organs in females and are often associated with anomalies of these structures^3^ as in OHVIRA syndrome (obstructed hemivagina and ipsilateral renal anomaly). There are increasing reports showing that associated renal pathology in OHVIRA syndrome is not limited to renal agenesis but includes other renal anomalies.^4^ Hydrometrocolpos is often one of the initial findings in OHVIRA syndrome and is usually detected in the early postnatal period or even during prenatal screenings. We report a case of an ectopic single ureter that ended in an incomplete vaginal duplication. The late age at onset of the symptoms was a challenge to obtain the correct diagnosis and management plan, especially since ultrasonography in the neonatal period showed no evidence for hydrometrocolpos.

## 2 Case presentation

**Fig. 1:**
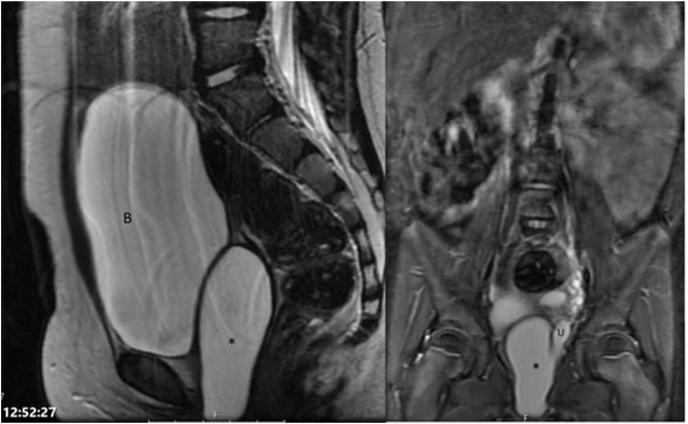
Sagittal and coronal MRU imaging demonstrating dilated left ectopic ureter (u) draining in a cystic structure inferior to the bladder (b) consistent with hydrocolpos (*).

**Fig. 2:**
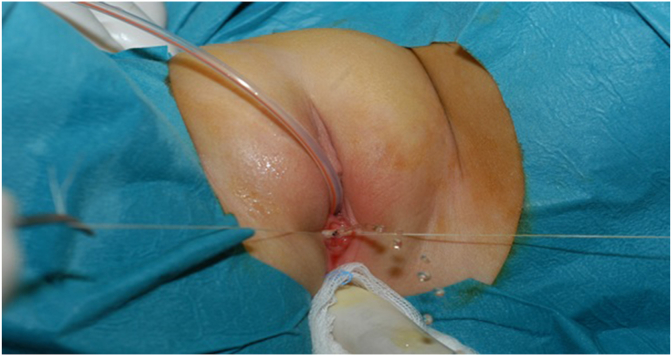
Intraoperative image. After partial resection of the vaginal wall, urine drained from the obstructed hemivagina.

**Fig. 3:**
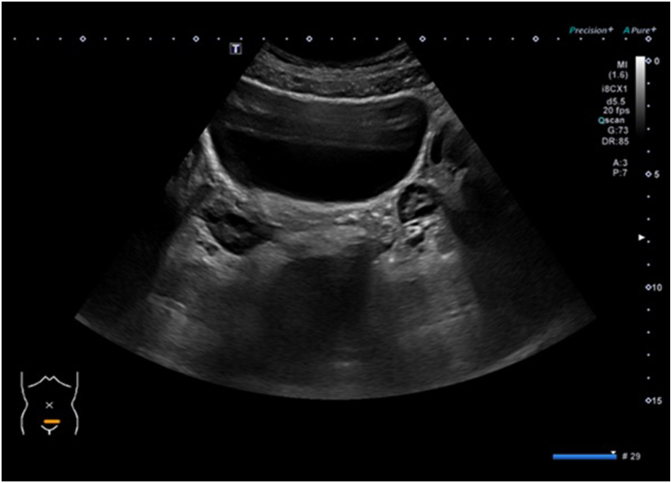
US image from postoperative follow-up showing no more cystic structure behind the bladder.

A 5-year-old continent girl was referred to our hospital for evaluation of lower abdominal pain. The girl had been in constant pain for the last few months, which worsened in the last few weeks. Prenatally, a multicystic dysplastic left kidney was diagnosed. A DMSA scan at 1 year of age showed no function of this kidney, while the ultrasonography showed no other abnormalities in the pelvic organs. Vesicoureteral reflux (VUR) was excluded by a voiding cystourethrogram (VCUG). On presentation, the initial physical examination showed normal external genitalia without any mass effect in the vagina, atresia of the hymen, or any vaginal discharge. Several blood and urine tests were performed and showed normal kidney function and no evidence of infection. Renal and pelvic ultrasound and later MR Urography (MRU) (Fig. 1) demonstrated a dilated ectopic left ureter ending in a large hydrocolpos but no evidence of renal tissue/function on the left side. Surgical intervention was indicated. Cystouretheroscopy showed a normal urethra, normal bladder urothelium and right ureteral orifice, whereas the left ureteral orifice was absent. Vaginoscopy showed a normal hymen and confirmed a vaginal duplication with an obstructed left hemivagina. The vaginal septum was partially excised and fluid consistent with urine was drained (Fig. 2). Vaginoscopy of the left hemivagina showed the portio and the orifice of the left ectopic ureter. No portio was identified in the right hemivagina. Laparoscopic nephroureterectomy was then performed. Histology confirmed a tiny dysplastic left kidney. Postoperative Ultrasonography demonstrated a normal vagina and no more cystic dilatation (Fig. 3). The girl no longer had abdominal pain and had no urological complaints at follow-up visits 6 months, 2 and 4 years post-operative.

## 3 Discussion

Hydrometrocolpos is a rare condition with fluid accumulation in the uterus and vagina characterized by a dilated, fluid-filled vaginal cavity due to a congenital malformation. In the majority of cases abdominal distension, abdominal mass, urinary obstruction and even respiratory distress occur during the neonatal period.^5^ Hematometrocolpos can also occur young girls, typically during puberty due to an imperforate hymen or OHVIRA syndrome.^4^ A hydrometrocolpos and other associated malformations can be diagnosed by prenatal ultrasound, although differentiation from a full bladder or other cystic abdominal masses, such as an ovarian cyst can be difficult. Delayed diagnosis and treatment of hydrometrocolpos in the neonatal period, especially in cases associated with cloacal malformations, can lead to serious complications.^5^ If undiagnosed, an obstructed vagina can present in young adulthood with dysmenorrhea, abdominal pain or even infertility. While an ectopic ureter is rarely diagnosed in a single renal system, most ectopic ureters are seen in the upper pole of a duplex kidney. In addition, ectopic ureters are associated with poorly functioning kidneys. In females, ectopic ureters often end distal to the external urinary sphincter of the urethra, resulting in incontinence, typically with constant dribbling.

Depending on the extent of the dysplasia, dysplastic kidneys produce more or less urine. In a multicystic dysplastic kidney, urine production may be completely absent. In our case, however, the highly dysplastic kidney still produced enough urine to lead to a symptomatic hydrometrocolpos at the age of 5 years.

As suggested by Schlomer et al.,^4^ OHVIRA syndrome should include not only ipsilateral renal agenesis but any form of renal dysplasia. Therefore, we recommend an ultrasonography of the internal Genitalia in infancy for all girls with unilateral renal dysplasia or agenesis. During mini puberty, the uterus is stimulated and an obstructed hemivagina, filled with mucus or urine, can often be diagnosed and treated early. If the findings are unremarkable, the parents should be informed about the possible presence of OHVIRA syndrome, with the recommendation of a pediatric gynecological at the onset of the puberty.

In our particular case, it was important to think of OHVIRA syndrome in order to set up a correct treatment plan. During puberty, we will monitor the girl closely to detect and treat any obstruction of the genital outflow tract at any early stage.

## 4 Conclusion

Late presentation of a symptomatic hydrometrocolpos is uncommon. It is important to remember that not only renal agenesis but also dysplasia or multicystic kidney, may be part of an OHVIRA syndrome. In girls with unilateral renal dysplasia, a duplication of the internal genitalia must always be considered.
